# Suboptimally controlled asthma in patients treated with inhaled ICS/LABA: prevalence, risk factors, and outcomes

**DOI:** 10.1038/s41533-023-00336-9

**Published:** 2023-05-08

**Authors:** Shiyuan Zhang, John White, Alyssa Goolsby Hunter, David Hinds, Andrew Fowler, Frances Gardiner, David Slade, Sharanya Murali, Wilhelmine Meeraus

**Affiliations:** 1 GSK, Collegeville, PA USA; 2 Optum, Eden Prairie, MN USA; 3 GSK, Brentford, Middlesex UK; 4 GSK, Research Triangle Park, NC USA

**Keywords:** Asthma, Outcomes research

## Abstract

This observational claims-linked survey study assessed the prevalence of and risk factors for suboptimal asthma control and healthcare utilization in adults with asthma receiving fixed-dose combination (FDC) inhaled corticosteroid/long-acting β_2_-agonist (ICS/LABA). Commercially insured adults from the Optum Research Database were invited to complete the Asthma Control Test (ACT) and Asthma Control Questionnaire-6 (ACQ-6). Among participants (*N* = 428), 36.4% (ACT-assessed) and 55.6% (ACQ-6-assessed) had inadequately controlled asthma. Asthma-related quality of life was worse and asthma-related healthcare resource utilization was higher in poorly controlled asthma. Factors associated with ACT-defined suboptimal asthma control in multivariate analysis included: frequent short-acting β_2_-agonist (SABA) use, asthma-related outpatient visits, lower treatment adherence, and lower education levels. During follow-up, factors associated with asthma exacerbations and/or high SABA use included: inadequately controlled asthma (ACT-assessed), body mass index ≥30 kg/m^2^, and high-dose ICS/LABA. Approximately 35–55% of adults with asthma were inadequately controlled despite FDC ICS/LABA; poor control was associated with worse disease outcomes.

## Introduction

Long-term goals for asthma management include achieving good control of asthma and minimizing the risk of asthma-related complications such as exacerbations^1^. Global Initiative for Asthma (GINA) guidelines recommend stepwise treatment escalation to achieve symptom control^1^. Combination therapy with an inhaled corticosteroid (ICS) and a long-acting β_2_-agonist (LABA) is a recommended option for daily maintenance therapy in patients whose asthma symptoms are not controlled on daily ICS or as-needed low-dose ICS-formoterol^1^.

Despite the widespread availability of different treatment options and guideline recommendations, asthma control remains suboptimal in a proportion of patients^2–8^. Estimates of the frequency of suboptimal asthma symptom control range from 50 to 70% in the United States (US)^3–5^, and 50 to 57% in Europe^6,7^, with variation due to differences in definitions for asthma control, healthcare settings, and populations^2–8^. Importantly, ~30–50% of patients with asthma remain inadequately controlled despite adherence to ICS/LABA therapy^9,10^. Compared with patients with optimal asthma control, suboptimal control of asthma is associated with increased exacerbation frequency, healthcare resource utilization (HCRU) and associated medical costs^2,4,8,9,11^, as well as lower health-related quality of life (HRQoL) and greater work impairment^8,9,12^. Consequently, the total economic burden associated with uncontrolled asthma among adolescents and adults in the US is projected to reach $963.5 billion over the next 20 years^13^. Therefore, achieving and maintaining asthma symptom control is essential for disease management to reduce the burden on both patients and healthcare resources.

Several validated instruments are available to assess asthma control, including the Asthma Control Test (ACT)^14^ and the Asthma Control Questionnaire (ACQ)^15–17^. There is no clear consensus as to which of these measures is more useful for the assessment of uncontrolled asthma, with a systematic review concluding that there is a strong and consistent correlation between ACT and ACQ scores^18^. There are limited studies evaluating how well these patient-reported outcome (PRO) instruments correlate with real-world asthma treatment practices and outcomes^18,19^. Previous survey-based studies have used the ACT to assess asthma control without stratifying patients by asthma maintenance treatment, an important factor associated with asthma control^20,21^. Additionally, real-world retrospective studies have used surrogate measures of asthma control, including short-acting β_2_-agonist (SABA) use, change in maintenance medication, and exacerbations^22–24^. There is a paucity of current real-world data describing asthma control among patients treated with fixed-dose combination (FDC) ICS/LABA therapy in the US^9,25^.

The objectives of this study were to determine the prevalence of suboptimal asthma control as assessed by the ACT and the ACQ in patients in the US treated with FDC ICS/LABA, to describe the impact of suboptimal asthma control on patients and HCRU, to identify risk factors for suboptimal control, and to determine the impact of suboptimal asthma control on the future risk of asthma outcomes.

## Results

### Study population

Of the 2250 patients who met claims-based sample identification criteria and were invited to participate in the study, 428 patients met the eligibility criteria and were included in the survey sample (Supplementary Fig. 1). The survey response rate, as per AAPOR formulas was 22.2%. The mean (standard deviation [SD]) patient age was 49.8 (12.0) years, 286 (66.8%) of patients were female, and the mean (SD) age at asthma diagnosis was 23.9 (18.2) years (Table 1). In addition, 377 patients had ≥6 months of claims data during the follow-up period and met all eligibility criteria for the follow-up study population.

**Table 1:** Patient demographics and clinical characteristics during the baseline period according to ACT-assessed asthma control.

Overall survey cohort^a^				
	Overall^b^ *N* = 428	ACT-assessed level of asthma control		
		Poorly controlled *n* = 62	Somewhat controlled *n* = 94	Controlled *n* = 272
Age (years)^c^, mean (SD)	49.8 (12.0)	48.6 (11.4)	49.8 (12.4)	50.1 (12.1)
Female, *n* (%)	286 (66.8)	47 (75.8)	66 (70.2)	173 (63.6)
Race, *n* (%)				
American Indian or Alaskan Native	7 (1.6)	1 (1.6)	4 (4.3)	2 (0.7)
Asian	4 (0.9)	0 (0.0)	1 (1.1)	3 (1.1)
Black or African American	26 (6.1)	8 (12.9)	6 (6.4)	12 (4.4)
Native Hawaiian or Pacific Islander	2 (0.5)	0 (0.0)	0 (0.0)	2 (0.7)
White	378 (88.3)	47 (75.8)	86 (91.5)	245 (90.1)
Other race	19 (4.4)	8 (12.9)	1 (1.1)	10 (3.7)
Missing	2 (0.5)	0 (0.0)	0 (0.0)	2 (0.7)
Hispanic or Latino ethnicity, *n* (%)				
Yes	39 (9.18)	10 (16.13)	5 (5.32)	24 (8.92)
No	386 (90.82)	52 (83.87)	89 (94.68)	245 (91.08)
Missing	3 (0.0)	0 (0.0)	0 (0.0)	3 (0.0)
Smoking status, *n* (%)	*n* = 423	*n* = 61	*n* = 93	*n* = 269
Current smoker	13 (3.1)	4 (6.6)	3 (3.2)	6 (2.2)
Former smoker	119 (28.1)	19 (31.2)	27 (29.0)	73 (27.1)
Never smoked, but I live with someone who smokes	34 (8.0)	5 (8.2)	11 (11.8)	18 (6.7)
Never smoked, no one in my household smokes	257 (60.8)	33 (54.1)	52 (55.9)	172 (63.9)
BMI (kg/m^2^), mean (SD)	29.9 (6.9)	31.2 (7.1)	30.8 (8.3)	29.3 (6.2)
BMI category (kg/m^2^), *n* (%)	*n* = 425	*n* = 61	*n* = 94	*n* = 270
Underweight (<18.5)	4 (0.9)	0 (0.0)	1 (1.1)	3 (1.1)
Normal (18.5–24.9)	105 (24.7)	14 (23.0)	21 (22.3)	70 (25.9)
Overweight (25–29.9)	133 (31.3)	16 (26.2)	28 (29.8)	89 (33.0)
Obese (≥30)	183 (43.1)	31 (50.8)	44 (46.8)	108 (40.0)
Marital status, *n* (%)				
Single, never married	58 (13.6)	7 (11.3)	12 (12.8)	39 (14.3)
Living with partner	30 (7.0)	6 (9.7)	6 (6.4)	18 (6.6)
Separated	6 (1.4)	1 (1.6)	1 (1.1)	4 (1.5)
Married	288 (67.3)	43 (69.4)	63 (67.0)	182 (66.9)
Divorced	39 (9.1)	4 (6.5)	9 (9.6)	26 (9.6)
Widowed	7 (1.6)	1 (1.6)	3 (3.2)	3 (1.1)
Highest level of education completed, *n* (%)	*n* = 427	*n* = 62	*n* = 93	*n* = 272
Some college but no degree or lower	144 (33.7)	25 (40.3)	38 (40.9)	81 (29.8)
2-year college or higher	283 (66.3)	37 (59.7)	55 (59.1)	191 (70.2)
Place of residence, *n* (%)	*n* = 426	*n* = 60	*n* = 94	*n* = 272
Urban/city	128 (30.1)	18 (30.0)	29 (30.9)	81 (29.8)
Suburban	224 (52.6)	29 (48.3)	42 (44.7)	153 (56.3)
Rural	74 (17.4)	13 (21.7)	23 (24.5)	38 (14.0)
Age (years) at asthma diagnosis, mean (SD)	*n* = 424	*n* = 62	*n* = 93	*n* = 269
	23.9 (18.2)	24.1 (19.4)	24.7 (18.0)	23.6 (18.1)
CCI score, mean (SD)	1.2 (0.8)	1.5 (1.1)	1.1 (0.5)	1.2 (0.8)
Selected comorbidities^d^, *n* (%)				
Asthma-related allergies	221 (51.6)	26 (41.9)	51 (54.3)	144 (52.9)
URTI	91 (21.3)	21 (33.9)	23 (24.5)	47 (17.3)
Allergies with URTI	89 (20.8)	18 (29.0)	18 (19.2)	53 (19.5)
Anxiety	77 (18.0)	13 (21.0)	18 (19.2)	46 (16.9)
Depression	70 (16.4)	13 (21.0)	14 (14.9)	43 (15.8)
Pneumonia	14 (3.3)	6 (9.7)	2 (2.1)	6 (2.2)
Any asthma exacerbation^e^, *n* (%)	75 (17.5)	19 (30.7)	17 (18.1)	39 (14.3)

*ACT* asthma control test, *BMI* body mass index, *CCI* Charlson comorbidity index, *ED* emergency department, *SD* standard deviation, *URTI* upper respiratory tract infection.

^a^Defined as 12 months up to and including survey (index) date. Please refer to Supplementary Table 2 for information on sources of specific data (patient survey and claims analysis).

^b^Overall study cohort: patients with survey data and claims data for the 12-month baseline period.

^c^Claims-based age calculated as of 2019.

^d^Five most common comorbidities, in addition to pneumonia.

^e^Hospitalization-, ED-, or corticosteroid-defined exacerbation, based on medical and pharmacy claims and asthma diagnosis code.

### Asthma control

**Fig. 1:**
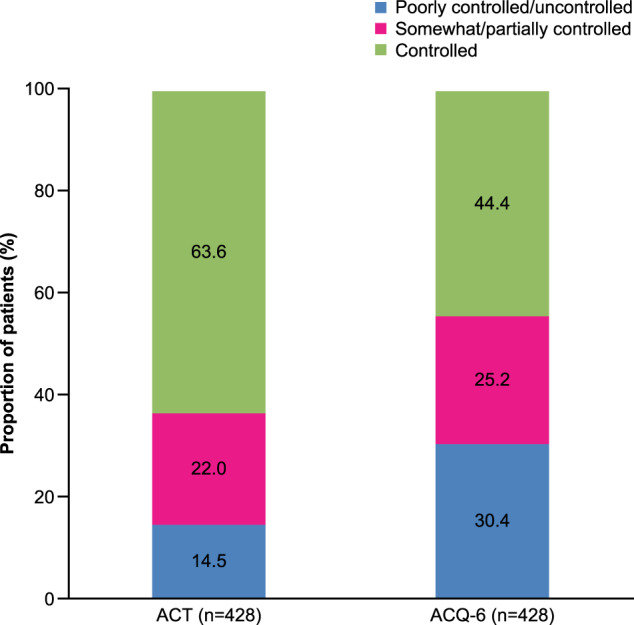
Level of patient-reported asthma control by ACT* or ACQ-6^†^ score. Overall study population. Overall study cohort: patients with survey data and claims data for the 12-month baseline period. *ACT recall: 4 weeks; score thresholds: <16, poorly controlled; 16–19, somewhat controlled; >19, controlled; ^†^ACQ-6 recall: 1 week; score thresholds: ≥1.50, uncontrolled; >0.75–<1.50, partially controlled; ≤0.75, controlled. ACT asthma control test, ACQ asthma control questionnaire.

Overall, 36.4% (*n* = 156) of patients did not have controlled asthma as assessed by the ACT; 14.5% (*n* = 62) of patients had poorly controlled asthma and 22.0% (*n* = 94) of patients had somewhat controlled asthma, with 63.6% (*n* = 272) of patients having controlled asthma (Fig. 1). In total, 311 (72.7%) patients reported shortness of breath at least once a week and 166 (38.8%) patients reported using their rescue inhaler at least two or three times per week in the previous 4 weeks on the ACT. Patients with ACT-assessed poorly controlled asthma tended to have higher mean BMI and were more likely to be female and former/current smokers relative to patients with controlled asthma (Table 1).

According to the ACQ-6, 55.6% of patients did not have controlled asthma; 30.4% (*n* = 130) of patients had uncontrolled asthma, 25.2% (*n* = 108) of patients had partially controlled asthma, while 44.4% (*n* = 190) patients had controlled asthma (Fig. 1 and Supplementary Table 1). There was a high degree of concordance between ACT and ACQ-6 scores in identifying asthma control (Spearman correlation coefficient −0.84) (Table 2 and Supplementary Fig. 2).

**Table 2:** Concordance between ACT- and ACQ-6-assessed asthma control, stratified by ACT level of control.

Overall study cohort				
	Overall *N* = 428	ACT^a^-assessed level of asthma control		
		Poorly controlled *n* = 62 (14.5%)	Somewhat controlled *n* = 94 (22.0%)	Controlled *n* = 272 (63.6%)
ACQ-6 score^b^, mean (SD)	1.06 (0.85)	2.33 (0.76)	1.49 (0.56)	0.61 (0.52)
ACQ-6 levels of asthma control^b^, *n* (%)				
Uncontrolled	130 (30.4)	56 (90.3)	50 (53.2)	24 (8.8)
Partially controlled	108 (25.2)	4 (6.5)	35 (37.2)	69 (25.4)
Controlled	190 (44.4)	2 (3.2)	9 (9.6)	179 (65.8)
On average, during the past week, how many times were you woken by your asthma during the night? *n* (%)				
Never	244 (57.0)	4 (6.5)	32 (34.0)	208 (76.5)
Hardly ever	99 (23.1)	14 (22.6)	37 (39.4)	48 (17.7)
A few times	63 (14.7)	28 (45.2)	21 (22.3)	14 (5.2)
Several times	10 (2.3)	6 (9.7)	3 (3.2)	1 (0.4)
Many times	7 (1.6)	6 (9.7)	0 (0.0)	1 (0.4)
A great many times	5 (1.2)	4 (6.5)	1 (1.1)	0 (0.0)
Unable to sleep because of asthma	0 (0.0)	0 (0.0)	0 (0.0)	0 (0.0)
On average, during the past week, how bad were your asthma symptoms when you woke up in the morning? *n* (%)				
No symptoms	151 (35.3)	2 (3.2)	11 (11.7)	138 (50.7)
Very mild symptoms	138 (32.2)	11 (17.7)	36 (38.3)	91 (33.5)
Mild symptoms	92 (21.5)	21 (33.9)	31 (33.0)	40 (14.7)
Moderate symptoms	45 (10.5)	26 (41.9)	16 (17.0)	3 (1.1)
Quite severe symptoms	2 (0.5)	2 (3.2)	0 (0.0)	0 (0.0)
Severe symptoms	0 (0.0)	0 (0.0)	0 (0.0)	0 (0.0)
Very severe symptoms	0 (0.0)	0 (0.0)	0 (0.0)	0 (0.0)
In general, during the past week, how limited were you in your activities because of your asthma? *n* (%)				
Not limited at all	198 (46.3)	10 (16.1)	21 (22.3)	167 (61.4)
Very slightly limited	111 (25.9)	7 (11.3)	37 (39.4)	67 (24.6)
Slightly limited	74 (17.3)	22 (35.5)	22 (23.4)	30 (11.0)
Moderately limited	36 (8.4)	17 (27.4)	12 (12.8)	7 (2.6)
Very limited	8 (1.9)	5 (8.1)	2 (2.1)	1 (0.4)
Extremely limited	1 (0.2)	1 (1.6)	0 (0.0)	0 (0.0)
Totally limited	0 (0.0)	0 (0.0)	0 (0.0)	0 (0.0)
In general, during the past week, how much shortness of breath did you experience because of your asthma? *n* (%)				
None	99 (23.1)	0 (0.0)	3 (3.2)	96 (35.3)
A very little	136 (31.8)	4 (6.5)	21 (22.3)	111 (40.8)
A little	99 (23.1)	16 (25.8)	36 (38.3)	47 (17.3)
A moderate amount	72 (16.8)	27 (43.6)	29 (30.9)	16 (5.9)
Quite a lot	17 (4.0)	10 (16.1)	5 (5.3)	2 (0.7)
A great deal	4 (0.9)	4 (6.5)	0 (0.0)	0 (0.0)
A very great deal	1 (0.2)	1 (1.6)	0 (0.0)	0 (0.0)
In general, during the past week, how much time did you wheeze? *n* (%)				
Never	140 (32.7)	4 (6.5)	9 (9.6)	127 (46.7)
Hardly any of the time	120 (28.0)	5 (8.1)	23 (24.5)	92 (33.8)
A little of the time	113 (26.4)	20 (32.3)	49 (52.1)	44 (16.2)
A moderate amount of time	40 (9.4)	24 (38.7)	9 (9.6)	7 (2.6)
A lot of the time	8 (1.9)	3 (4.8)	3 (3.2)	2 (0.7)
Most of the time	4 (0.9)	3 (4.8)	1 (1.1)	0 (0.0)
All of the time	3 (0.7)	3 (4.8)	0 (0.0)	0 (0.0)
On average, during the past week, how many puffs/inhalations of short-acting bronchodilator (e.g., Ventolin/Bricanyl) have you used each day? *n* (%)				
	*n* = 425	*n* = 60	*n* = 94	*n* = 271
None	180 (42.4)	2 (3.3)	11 (11.7)	167 (61.6)
1–2 puffs/inhalations most days	171 (40.2)	22 (36.7)	59 (62.8)	90 (33.2)
3–4 puffs/inhalations most days	50 (11.8)	18 (30.0)	18 (19.2)	14 (5.2)
5–8 puffs/inhalations most days	21 (4.9)	15 (25.0)	6 (6.4)	0 (0.0)
9–12 puffs/inhalations most days	2 (0.5)	2 (3.3)	0 (0.0)	0 (0.0)
13–16 puffs/inhalations most days	0 (0.0)	0 (0.0)	0 (0.0)	0 (0.0)
>16 puffs/inhalations most days	1 (0.2)	1 (1.7)	0 (0.0)	0 (0.0)

Overall study cohort: patients with survey data and claims data for the 12-month baseline period.

*ACQ-6* asthma control questionnaire-6, *ACT* asthma control test, *SABA* short-acting β_2_-agonist.

^a^ACT recall: 4 weeks; score thresholds: <16, poorly controlled; 16–19, somewhat controlled; >19, controlled.

^b^ACQ-6 recall: 1 week; score thresholds: ≥1.5, uncontrolled; >0.75–<1.5, partially controlled; ≤0.75, controlled. Three subjects had missing responses to the ACQ question on SABA use; however, they were not excluded from the analysis. All other ACQ items were required.

### Asthma-related HRQoL and health status

HRQoL, as measured by the mini-AQLQ, was worse in patients with poorly controlled asthma (mean [SD] 3.81 [0.96]) compared with controlled asthma (5.89 [0.84]) as measured by the ACT (Table 3 and Supplementary Table 2). Similar results were observed with the ACQ-6, where patients with uncontrolled asthma had a lower mean (SD) asthma-related HRQoL as measured by the mini-AQLQ (4.19 [0.99]) versus those with controlled asthma (6.16 [0.67]).

**Table 3:** Asthma-related quality of life and health status stratified by ACT level of control.

Overall study cohort				
	Overall *N* = 428	ACT-assessed level of asthma control		
		Poorly controlled *n* = 62	Somewhat controlled *n* = 94	Controlled *n* = 272
Mini-AQLQ^a^	*n* = 419	*n* = 60	*n* = 93	*n* = 266
Overall score, mean (SD)	5.34 (1.17)	3.81 (0.96)	4.73 (0.89)	5.89 (0.84)
EQ-5D-3L	*n* = 410	*n* = 55	*n* = 91	*n* = 264
VAS^b^, mean (SD)	77.31 (13.97)	68.38 (15.81)	72.79 (14.70)	80.73 (11.95)
Index (utility) score^c^, mean (SD)	*n* = 423	*n* = 61	*n* = 93	*n* = 269
	0.87 (0.14)	0.80 (0.16)	0.84 (0.13)	0.90 (0.13)

Overall study cohort: patients with survey data and claims data for the 12-month baseline period.

*ACT* asthma control test, *ACQ-6* asthma control questionnaire-6, *EQ-5D-3L* EuroQol group 5 dimension health status measure, 3-level, *mini-AQLQ* Asthma quality of life questionnaire, short version, *SD* standard deviation, *VAS* visual analog scale.

^a^Each mini-AQLQ question is answered on a scale from 1 to 7 and covers a 2-week recall period, with lower scores indicating greater impairment.

^b^VAS ranges from 0 to 100, where 100 is the best health imaginable and 0 is the worst health imaginable.

^c^EQ-5D-3L health states were converted into a single summary index score by applying weights to each of the levels in each dimension using the time trade-off valuation technique and is specific to the US population. An index score of 1 represents full health, with lower scores indicating worse health status on the day the respondent is answering.

Mean (SD) health status as assessed by the EQ-5D-3L index utility score was also worse in patients with ACT-assessed poorly controlled asthma (0.80 [0.16]) compared with controlled asthma (0.90 [0.13]) (Table 3 and Supplementary Table 1). This was consistent with the ACQ-6 findings, where the mean (SD) EQ-5D-3L index utility score was 0.81 (0.15) for patients with uncontrolled asthma and 0.91 (0.13) for patients with controlled asthma.

### Medication use and adherence

The proportion of patients with optimal adherence (FDC ICS/LABA medication possession ratio [MPR] during the 12-month baseline period ≥0.8) was lower among those classed as having poorly controlled versus controlled asthma by the ACT (35.6% [*n* = 21] vs 46.7% [*n* = 126], respectively) (Table 4). Similar trends were seen for patients with MPR ≥0.5. In addition, the proportion of patients with a PDC over the 12-month baseline period ≥0.8 or ≥0.5% for FDC ICS/LABA therapy was lower for patients with poorly controlled and somewhat controlled asthma compared with controlled asthma (Table 4).

**Table 4:** Medication and adherence stratified by ACT level of control.

Overall study cohort				
	Overall *N* = 428	ACT-assessed level of asthma control		
		Poorly controlled *n* = 62	Somewhat controlled *n* = 94	Controlled *n* = 272
SABA use^a^, *n* (%)	271 (63.3)	49 (79.0)	73 (77.7)	149 (54.8)
SABA canisters, *n* (%)				
≤6 per year	386 (90.2)	45 (72.6)	82 (87.2)	259 (95.2)
>6 per year	42 (9.8)	17 (27.4)	12 (12.8)	13 (4.8)
ICS/LABA dose category, *n* (%)				
Low	182 (42.5)	15 (24.2)	41 (43.6)	126 (46.3)
Medium	168 (39.3)	33 (53.2)	37 (39.4)	98 (36.0)
High	78 (18.2)	14 (22.6)	16 (17.0)	48 (17.7)
Maintenance OCS use, *n* (%)	14 (3.3)	3 (4.8)	2 (2.1)	9 (3.3)
Adherence to FDC ICS/LABA therapy, *n* (%)				
	*n* = 422	*n* = 59	*n* = 93	*n* = 270
MPR ≥0.8	181 (42.9)	21 (35.6)	34 (36.6)	126 (46.7)
MPR ≥0.5	314 (74.4)	38 (64.4)	64 (68.8)	212 (78.5)
PDC for FDC ICS/LABA, mean (SD)	0.54 (0.24)	0.46 (0.24)	0.51 (0.24)	0.57 (0.23)
PDC categories, *n* (%)				
≥0.8	81 (18.9)	7 (11.3)	13 (13.8)	61 (22.4)
≥0.5	225 (52.6)	25 (40.3)	47 (50.0)	153 (56.3)

Overall study population: patients with survey data and claims data for the 12-month baseline period.

*ACT* asthma control test, *FDC* fixed-dose combination, *ICS* inhaled corticosteroid, *LABA* long-acting β_2_-agonist, *MPR* medication possession ratio, *OCS* oral corticosteroid, *PDC* proportion of days covered, *SABA* short-acting β2-agonist, *SD* standard deviation.

^a^SABA rescue medication fills from pharmacy claims in a 12-month baseline period (including index date).

In total, 63.3% (*n* = 271) of patients had ≥1 claim for a SABA during the baseline period, and this proportion was higher in patients with ACT-assessed poorly controlled asthma compared with patients with controlled asthma (Table 4). In total, 90.2% (*n* = 386) of patients were using ≤6 SABA canisters per year. A higher proportion of patients with ACT-assessed poorly controlled asthma were using medium- (53.2% [*n* = 33]) and high-dose (22.6% [*n* = 14]) ICS/LABA therapy compared with patients with controlled asthma (36.0% [*n* = 98] and 17.7% [*n* = 48]) (Table 4).

### HCRU and costs

Patients with ACT-assessed poorly controlled asthma had numerically higher baseline asthma-related HCRU (ambulatory, outpatient, and emergency department [ED] visits) compared with patients with better asthma control (Table 5). Median HCRU costs were generally similar between the different ACT-assessed asthma control groups, for both all-cause and asthma-related costs (Table 5).

**Table 5:** All-cause and asthma-related HCRU and costs stratified by ACT level of control.

Overall study cohort				
	Overall *N* = 428	ACT-assessed level of asthma control		
		Poorly controlled *n* = 62	Somewhat controlled *n* = 94	Controlled *n* = 272
All-cause HCRU, *n* (%)				
Ambulatory visit	427 (99.8)	62 (100)	94 (100)	271 (99.6)
Office visit	427 (99.8)	62 (100)	94 (100)	271 (99.6)
Outpatient visit	259 (60.5)	43 (69.4)	58 (61.7)	158 (58.1)
ED visit	139 (32.5)	25 (40.3)	24 (25.5)	90 (33.1)
Asthma-related HCRU, *n* (%)				
Ambulatory visit	262 (61.2)	43 (69.4)	57 (60.6)	162 (59.6)
Office visit	253 (59.1)	36 (58.1)	57 (60.6)	160 (58.8)
Outpatient visit	32 (7.5)	14 (22.6)	8 (8.5)	10 (3.7)
ED visit	11 (2.6)	6 (9.7)	1 (1.1)	4 (1.5)
All-cause HCRU costs^a^ ($), median (IQ range)				
Total costs (medical + pharmacy)	7080 (4380, 13123)	7543 (4025, 14305)	6128 (4173, 11566)	7298 (4736, 14371)
Pharmacy costs	3585 (2335, 5344)	3604 (1992, 5702)	3295 (2418, 5213)	3684 (2371, 5635)
Medical costs	2837 (1169, 7837)	3554 (1596, 9014)	2356 (875, 6718)	2859 (1222, 7979)
Ambulatory costs	2198 (964, 5976)	2470 (1073, 7730)	2141 (787, 5338)	2183 (1017, 5335)
Outpatient visit costs	294 (0, 2481)	544 (0, 3480)	308 (0, 2603)	228 (0, 2300)
ED costs	0 (0, 121)	0 (0, 270)	0 (0, 0)	0 (0, 127)
Asthma-related HCRU costs^a^ ($), median (IQ range)				
Total costs (medical + pharmacy)	3021 (1914, 4138)	2708 (1611, 4133)	2899 (1952, 4160)	3086 (1977, 4138)
Pharmacy costs	2729 (1774, 3877)	2230 (1142, 3656)	2729 (1818, 3871)	2885 (1826, 3917)
Medical costs	102 (0, 267)	149 (0, 490)	81 (0, 263)	97 (0, 259)
Ambulatory costs	101 (0, 266)	149 (0, 484)	81 (0, 263)	92 (0, 259)
Outpatient visit costs	0 (0, 0)	0 (0, 0)	0 (0, 0)	0 (0, 0)
ED costs	0 (0, 0)	0 (0, 0)	0 (0, 0)	0 (0, 0)

Overall survey cohort: patients with survey data and claims data for the 12-month baseline period.

*ACT* asthma control test, *ED* emergency department, *HCRU* healthcare resource utilization, *IQ* interquartile, *SD* standard deviation.

^a^Total costs were adjusted using the annual medical care component of the Consumer Price Index to reflect inflation to the year 2018.

### Risk factors for suboptimal asthma control

**Fig. 2:**
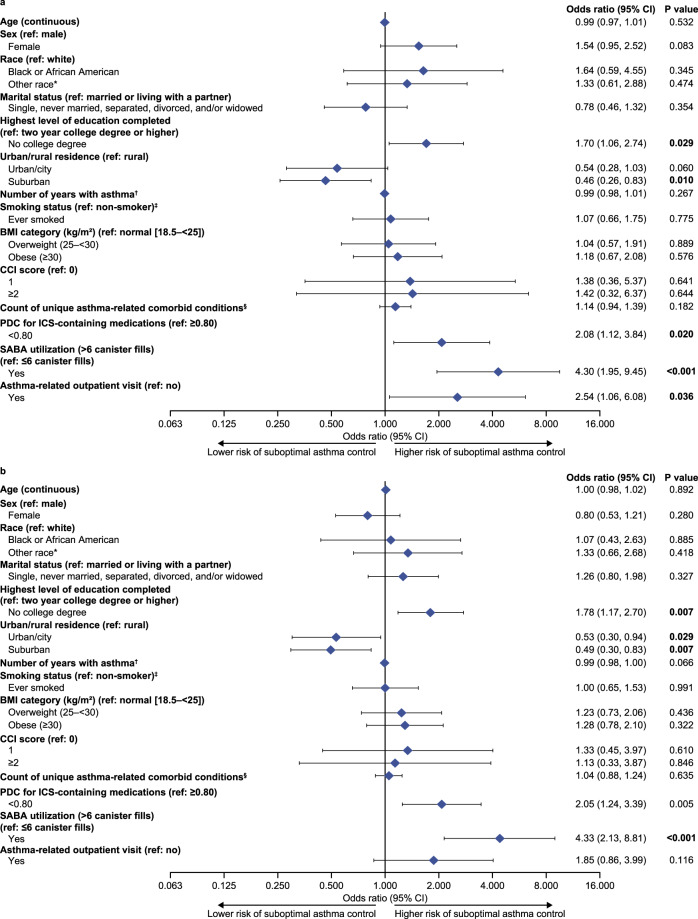
Multivariate analysis of potential factors associated with suboptimal asthma control. Measured by **a** ACT score and **b** ACQ-6 score (overall study population). Overall study population: patients with survey data and claims data for the 12-month baseline period. Missing values for race were categorized as “White” and for urban/rural residence as “suburban”; missing values for other variables were not imputed. *“Other race” included patients that self-reported their race as American Indian or Alaskan Native, Asian, Native Hawaiian or Pacific Islander, or Other race, as well as those that selected more than one response (i.e., multiracial); ^†^asthma duration was calculated by subtracting the age when asthma was first diagnosed by a doctor or other healthcare provider from the respondent’s current self-reported age; ^ǂ^the non-smoker category included patients who reported that they have never smoked and those who live with someone that smokes; ^§^presence of an asthma-related comorbid condition was defined as ≥1 claim with a diagnosis code for angina, cataract, myocardial infarction, pneumonia, upper respiratory tract infection, depression, anxiety, allergy, allergy/URTI combination, or type-2 diabetes mellitus. The potential range of unique asthma-related comorbid conditions was 0–10 (observed range: 0–6 conditions). ACT asthma control test, ACQ asthma control questionnaire, BMI body mass index, CCI Charlson comorbidity index, CI confidence interval, ICS inhaled corticosteroid, PDC proportion of days covered, SABA short-acting β_2_-agonist, URTI upper respiratory tract infection.

The multivariate analysis of potential factors associated with asthma control measured by the ACT found that, after adjustment for covariates, a lower level of education (odds ratio [OR] [95% CI]: 1.70 [1.06, 2.74], *p* = 0.029), PDC <0.80 for ICS-containing medications (2.08 [1.12, 3.84], *p* = 0.020), SABA use >6 canister fills in the previous 12 months (4.30 [1.95, 9.45], *p* < 0.001) and having made an asthma-related outpatient visit (2.54 [1.06, 6.08], *p* = 0.036) significantly increased the odds of suboptimal asthma control (Fig. 2a).

The corresponding multivariate analysis according to the ACQ-6 found that after adjustment for covariates, a lower level of education (OR [95% CI]: 1.78 [1.17, 2.70], *p* = 0.007), PDC <0.80 for ICS-containing medications (2.05 [1.24, 3.39], *p* = 0.005) and SABA use >6 canister fills (4.33 [2.13, 8.81], *p* < 0.001) increased the odds of suboptimal asthma (Fig. 2b). Both multivariate analyses found that, compared with a rural area, living in a suburban area was associated with greater asthma control (ACT score: 0.46 [0.26, 0.83], *p* = 0.010; ACQ-6 score: 0.49 [0.30, 0.83], *p* = 0.007), while living in an urban/city environment was also associated with greater asthma control as measured by the ACQ-6 (0.53 [0.30, 0.94], *p* = 0.029).

### Exacerbations, HCRU, and SABA canister fills during the follow-up period

During the 6-month follow-up period, exacerbations were reported in 6.3% (15/237) of patients with controlled asthma, according to the ACT, compared with 12.8% (11/86) patients with somewhat controlled asthma and 7.4% (4/54) patients with poorly controlled asthma (Table 6). HCRU and costs followed a similar pattern as observed in the baseline period, although the follow-up study cohort had fewer patients (Table 6). Among patients who had >4 SABA canister fills a year, there was a higher proportion with poorly controlled asthma (25.9% [14/54]), than with somewhat controlled (15.1% [13/86]), or controlled asthma (8.4% [20/237]) (Table 6).

**Table 6:** Asthma exacerbations, healthcare utilization, and healthcare costs during the 6-month follow-up period by ACT level of asthma control association.

Follow-up cohort				
	Overall *N* = 377	ACT-assessed level of asthma control		
		Poorly controlled *n* = 54	Somewhat controlled *n* = 86	Controlled *n* = 237
Any asthma exacerbation^a^ during the follow-up period, *n* (%)	30 (8.0)	4 (7.4)	11 (12.8)	15 (6.3)
Asthma exacerbation^a^ count in those with at least ≥1 exacerbation, mean (SD)	1.23 (0.50)	1.25 (0.50)	1.36 (0.67)	1.13 (0.35)
>4 SABA canisters per year (annualized)^b^, *n* (%)	47 (12.5)	14 (25.9)	13 (15.1)	20 (8.4)
Asthma-related HCRU, *n* (%)				
Ambulatory visit	123 (32.6)	20 (37.0)	29 (33.7)	74 (31.2)
Office visit	120 (31.8)	19 (35.2)	28 (32.6)	73 (30.8)
Outpatient visit	17 (4.5)	4 (7.4)	4 (4.7)	9 (3.8)
ED visit	3 (0.8)	1 (1.9)	0	2 (0.8)
Asthma-related HCRU costs^c^ ($), median (IQ range)				
Total costs (medical + pharmacy)	241 (112, 348)	223 (57, 367)	235 (87, 341)	256 (125, 348)
Pharmacy costs	234 (106, 331)	210 (55, 329)	204 (66, 317)	237 (116, 338)
Medical costs among patients with medical costs >$0	*n* = 125	*n* = 21	*n* = 30	*n* = 74
	29 (18, 64)	31 (23, 69)	39 (26, 64)	25 (15, 63)
Ambulatory costs among patients with ambulatory costs >$0	*n* = 121	*n* = 20	*n* = 29	*n* = 72
	29 (18, 64)	32 (24, 73)	41 (26, 64)	25 (16, 64)

Follow-up study cohort: patients from the overall study cohort who were also continuously enrolled for the 6-month follow-up period.

*ACT* asthma control test, *ED* emergency department, *HCRU* healthcare resource utilization, *IQ* interquartile, *SABA* short-acting β_2_-agonist, *SD* standard deviation.

^a^Hospitalization-, ED-, or corticosteroid-defined exacerbation, based on medical and pharmacy claims and asthma diagnosis code.

^b^Annualized count of SABA canisters was calculated by multiplying the number of canisters in the 6-month follow-up period by 2.

^c^Costs per patient per month; total costs were adjusted using the annual medical care component of the Consumer Price Index to reflect inflation to the year 2018.

### Impact of asthma control on future risk of exacerbations and high SABA use

**Fig. 3:**
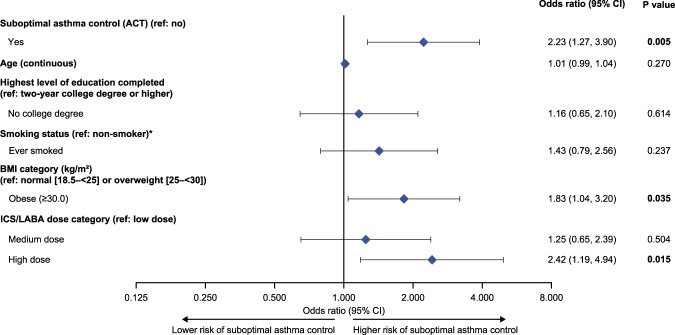
Multivariate analysis of potential factors associated with increased odds of an asthma exacerbation and/or high SABA use in the 6-month follow-up period. Follow-up study cohort. Follow-up study cohort: patients from the overall study cohort who were also continuously enrolled for the 6-month follow-up period. *The non-smoker category included patients who reported they have never smoked and those who live with someone that smokes. ACT asthma control test, BMI body mass index, CI confidence interval, ICS inhaled corticosteroid, LABA long-acting β_2_-agonist, SABA short-acting β_2_-agonist.

The final multivariate model evaluated factors associated with having increased odds of an asthma exacerbation and/or high SABA use during the 6-month follow-up period. This model found that having poorly or somewhat controlled asthma according to the ACT (OR [95% CI]: 2.23 [1.27, 3.90], *p* = 0.005), having a BMI ≥30 kg/m^2^ (1.83 [1.04, 3.20], *p* = 0.035), and receiving high-dose ICS/LABA therapy (2.42 [1.19, 4.94], *p* = 0.015) were associated with significantly increased odds of these events (Fig. 3).

## Discussion

In this US-based observational study of administrative claims data linked with cross-sectional survey data, ~35–55% of participants self-reported inadequately controlled asthma as assessed by the ACT or ACQ-6 despite receiving FDC ICS/LABA. The proportion of patients with higher rates of adherence to FDC ICS/LABA (MPR ≥0.8 or ≥0.5) was greater among patients classed as controlled by the ACT, although 11.3 and 40.3% of poorly controlled patients, according to the ACT, had PDC ≥0.8 and ≥0.5, respectively. These findings are broadly consistent with previous studies of asthma control conducted in the US among patients treated with ICS/LABA^3–5,8,9^.

Although the ACT classified more patients in the controlled category than the ACQ-6, there was a high degree of concordance between the ACT and ACQ-6, particularly in rating patients as uncontrolled, where 90% of patients with poorly controlled asthma on the ACT had uncontrolled asthma on the ACQ-6. This strong positive concordance between the ACT and ACQ-6 is in agreement with a previous literature review^18^.

The multivariate analyses found that lower levels of education, poor treatment adherence, and frequent SABA use were independently associated with uncontrolled asthma as assessed by both ACT and ACQ-6. This is consistent with a previous analysis of the ACCESS surveys, where treatment adherence and education level were predictive of poor asthma control, in addition to BMI, being a current smoker, and several other factors^26^. The multivariate analyses also suggested that living in a suburban area, which may be a proxy for access to care, socioeconomic status, or other factors that may improve asthma management, was associated with greater asthma control compared with living in a rural area, which differs from the results of a previous study^6^. During the 6-month follow-up period, the multivariate analysis found that suboptimal asthma control, in addition to a higher BMI, and high-dose ICS/LABA therapy, were independently associated with patients having increased odds of an asthma exacerbation and/or high SABA use. Accordingly, the risk of asthma exacerbations in patients with poorly or somewhat controlled asthma was twice that for patients with controlled asthma, highlighting the negative outcomes for patients with uncontrolled disease.

Both asthma-related HRQoL and overall health status were higher in patients with controlled asthma relative to patients with uncontrolled or poorly controlled asthma (ACT and ACQ-6). In the current study, patients with poor asthma control reported more frequent night-time awakenings than patients with better control, a factor which may contribute to lower HRQoL in patients with uncontrolled asthma^27^. In the 12 months prior to completing the survey, patients with asthma who were poorly controlled also had numerically higher asthma-related HCRU than patients with controlled disease. Similarly, in the 6-month follow-up period, exacerbation frequency, SABA use, and HCRU were numerically higher among patients with suboptimal asthma control compared with patients with controlled asthma; however, these differences should be interpreted with caution due to the relatively small sample sizes in the asthma control groups. These findings are consistent with previous studies^4,8,9,12^, highlighting the burden of uncontrolled asthma for patients, healthcare systems, and wider society.

The study is limited by characteristics associated with the use of administrative claims data, which were collected for the purposes of payment rather than research. Patients collecting pharmacy fills may not be taking their medication but may still be classified as adherent using MPR and PDC. Furthermore, the presence/absence of a diagnosis code is not definitive proof of the presence/absence of disease. To help mitigate these limitations, we included only those patients who self-reported current asthma maintenance treatment and who self-reported an asthma diagnosis by a healthcare provider. Limitations of the survey may include sampling, coverage, and measurement errors. The response rate was relatively low (22%), which also impacts the relatively small sample size of the follow-up cohort and which may limit the ability to identify risk factors for control and predictors of future exacerbations and SABA overuse. We did not, however, observe differences between the sample available for the baseline survey and the patients available for the follow-up analysis so the sample size is unlikely to have impacted the analysis. Additionally, the survey was administered in the springtime, meaning that seasonal variations in asthma control were not accounted for. Furthermore, patients who agree to participate in research studies such as the current one are likely to differ from those who decline to participate. It is important to note that the entire study was conducted pre-pandemic. A reduction in asthma exacerbations has been reported over the pandemic^28,29^, possibly because patients’ behavior with respect to adherence has been affected^30^. However, it is still unclear whether this change in behavior is permanent. Finally, the study included commercially insured adults being treated for asthma in the US, so the results may not apply to uninsured, older, or younger populations with asthma treated with ICS/LABA. Despite the limitations, our results were similar to those of a smaller cross-sectional survey of patients in the US receiving medium-to-high-dose ICS/LABA^9^.

Depending on the measure, over one-third to one-half of patients self-reported inadequately controlled asthma despite FDC ICS/LABA treatment. High levels of concordance were found between the two assessments of asthma control, the ACT and ACQ-6. Adherence appears to be associated with asthma control; however, 12% of patients with high adherence were still uncontrolled. Patients with poor asthma control in our study also had higher HCRU, poorer overall health status, and lower asthma-related HRQoL. The multivariate analyses found that lower levels of education, poor treatment adherence, and frequent SABA use were independently associated with uncontrolled asthma, and uncontrolled asthma was a risk factor for future asthma exacerbations and rescue medication use. Overall, this indicates a substantial burden to both patients and healthcare systems and highlights the unmet needs that remain among patients receiving FDC ICS/LABA maintenance therapy, which may be addressed by additional treatment options.

## Methods

### Study design and data sources

This was an observational study (HO-17-17252) of adults in the US with asthma treated with FDC ICS/LABA identified from the Optum Research Database (ORD). The ORD is a geographically diverse, de-identified research database comprising administrative claims, containing both medical and pharmacy information. It is built from a variety of geographic regions and employer groups, and thus it preserves a level of diversity and also represents the overall trend in commercial health plan coverage^31^. In 2018, ~19% of the US commercially enrolled population was represented in the ORD. The study was approved by the New England Institutional Review Board (IRB# 120190029) on 8 March 2019.

The sample identification period was 12 months (1 March 2018 to 28 February 2019), after which eligible patients were invited to participate via mail following the Dillman method^32^, with a survey fielding period of 8 weeks. As part of the Institutional Review Board (IRB) submission, Optum requested a waiver of documentation of informed consent. The study packet contained an IRB-approved informed consent statement, which did not require a signature. The consent form asked patients to return the study survey if they elected to participate in the study. Consent was implied when patients returned study materials, and signed consent was not obtained. Patients may have withdrawn consent at any time. Optum did not begin recruitment until IRB approval of all components of the study was obtained. Respondents were sent a $25 post-paid incentive for their study participation. Cross-sectional survey data from respondents was linked with medical and pharmacy claims data for the 12-month period prior to and including the survey completion date (baseline period); where available, data were also linked for the 6-month period following survey completion (follow-up period) (Supplementary Fig. 3).

### Sample identification

Eligible patients identified from the ORD were ≥18 years at the time of survey fielding, with ≥1 International Classification of Disease 10th edition Clinical Modification (ICD-10-CM) diagnosis code for asthma in any position during the 12-month sample identification period, 12 months of continuous enrollment including both commercial medical and pharmacy benefits, and ≥2 pharmacy claims for FDC ICS/LABA labeled for use with asthma (≥1 of these claims during the most recent 6 months of the sample identification period). Patients were excluded if they had an ICD-10-CM diagnosis code (i.e., ≥1 medical claim) for chronic obstructive pulmonary disease, cystic fibrosis, or interstitial lung diseases, or claims-based evidence of a diagnosis or treatment for lung cancer prior to or at the time of the survey fielding.

Patients identified by these claims-based sample criteria were grouped into one of three FDC ICS/LABA medication dose cohorts (patients with claims for only low and/or low-medium daily dose treatments [low-dose cohort], medium and/or medium-high daily dose treatments [medium-dose cohort], or only high daily dose treatments [high-dose cohort]; Supplementary Table 3). A random sample of 750 patients from each of these three medication dose cohorts was selected to receive the survey to ensure a range of asthma severity levels were included. Patients were excluded from the analytic sample if they did not return a complete survey (including evaluable ACT and ACQ-6), did not report a healthcare provider diagnosis of asthma, and/or did not report current asthma maintenance treatment. In addition to the claims criteria noted above, patients were also excluded from the final analytic sample if they did not have 12 months of continuous enrollment prior to and including the survey completion date (baseline). Patients who were disenrolled during the follow-up period were excluded from analyses that included the follow-up period.

### Measures

To assess the prevalence of suboptimal asthma control in patients treated with FDC ICS/LABA, risk factors associated with suboptimal asthma control, and the impact of suboptimal asthma control on patients and HCRU, the following measures were collected from patient surveys and administrative claims (from the ORD).

### Patient survey assessments

#### ACT-assessed asthma control

The five-item ACT was used to determine the patient’s level of asthma control^14^. The recall period is 4 weeks. Each of the items of the ACT is scaled on a 1–5 point scale with higher values indicative of better asthma control. The item response values of the ACT are summed to produce a single score that ranges from 5 (poor asthma control) to 25 (complete control of asthma). The minimum clinically important difference (MCID) is 3 points or greater. An ACT score was computed only if the patient provided a response to all five items. No processes are available for computing a summary score when one or more item responses are missing. In addition to the total score, the count and percent for all responses to each of the five items was reported.

#### ACQ-6 assessed asthma control

The six-item ACQ was used to determine the patient’s level of asthma control^15–17^. The recall period is 1 week. Each of the items of the ACQ is scaled from 0 (totally controlled) to 6 (severely uncontrolled). All items are equally weighted and the ACQ score is the mean of the six items. The MCID is 0.5 or greater. An ACQ score was computed only if the patient provided a response to all six items. No processes are available for computing a summary score when one or more item responses are missing. In addition to the total score, the count and percent for all responses to each of the six items were reported.

#### Asthma-related quality of life

The mini-AQLQ is a self-administered 15-item questionnaire that covers four domains: symptoms, activity limitation, emotional function, and environmental stimuli^33^. Each question is answered on a scale from 1–7, with lower scores indicating greater impairment. The recall period is 2 weeks prior to the test. An overall score and scores for each of the four domains are calculated. The overall score is the mean of all items, while the domain scores are the mean of the specific domain items. The MCID is 0.5 or greater.

#### General health status

The EQ-5D-3L was used to provide a descriptive profile and index value for health status^34^. The EQ-5D-3L contains two components. The first consists of five questions comprising dimensions of health (mobility, self-care, usual activities, pain/discomfort, and anxiety/depression) across three levels (no problems, some/moderate problems, and extreme problems). The second component consists of a visual analog scale (EQ VAS). The EQ VAS records the respondent’s self-rated health on a vertical scale with values from the worst health state imaginable (0) to the best health state imaginable (100). Levels of problems for each health state, EQ VAS scores and an EQ-5D-3L index (utility) score were calculated.

Full details of patient survey measures are included in Supplementary Table 4.

### Patient survey measures

For the study’s primary objective, participants were asked to assess their asthma control during the past 4 weeks using ACT^14^. ACT scores were used to categorize patients’ asthma as poorly controlled (ACT score <16), somewhat controlled (ACT score 16–19), or controlled (ACT score ≥20). In some analyses, ACT scores for the poorly and somewhat controlled (score of <16 and 16–19, respectively) groups were combined in a “suboptimal control” group, creating a binary ACT variable, with controlled (score ≥20) patients acting as the reference group. Participants also completed the ACQ-6^15–17^ based on their symptoms in the previous week, with total scores of ≥1.50 indicating uncontrolled asthma, >0.75–<1.50 indicating partially controlled asthma, and ≤0.75 indicating controlled asthma^17^. The ACQ-6 was used rather than the ACQ-7 in this study due to the absence of lung function in the ACQ-6, which is also absent from the ACT. Participants were also asked to complete the mini-Asthma Quality of Life Questionnaire (mini-AQLQ, recall period: 2 weeks)^33^ and the EuroQol Group 5 Dimension Health Status Measure, 3-level (EQ-5D-3L, recall period: today)^34^. The survey also collected data on sociodemographic characteristics, smoking behavior, current weight and height, and asthma treatment history.

### Claims measures

Claims data were used to assess geographic region, age, and sex (if missing from the patient survey), Charlson Comorbidity Index score^35^ (CCI; an assessment tool used to predict mortality by classifying or weighting comorbidities), asthma-related comorbidities, asthma treatment, MPR, the proportion of days covered (PDC), asthma exacerbations (hospitalization-, emergency department-, or corticosteroid-defined exacerbation, based on medical and pharmacy claims and asthma diagnosis code), HCRU, and costs. An updated CCI of 12 comorbidities was used^35^. Asthma-related comorbidities were defined as ≥1 claim with a diagnosis code for angina, cataract, myocardial infarction, pneumonia, upper respiratory tract infection, allergy, allergy/upper respiratory tract infection combination, type-2 diabetes mellitus, or a diagnosis or treatment code for depression or anxiety. ICS/LABA dose category was assigned according to the latest claim for an ICS/LABA medication prior to completing the survey based on the average daily dose and was medication-specific (Supplementary Table 5). MPR was calculated by summing the number of days supplied for an ICS/LABA for all but the last fill in the observation period and dividing by the number of days between the first and last refill. PDC was calculated by dividing the number of days on which medication was available (based on filled prescriptions) by the number of days during the observation period. Healthcare costs were computed as the combined health plan and patient-paid amounts.

### Sample size and statistical analyses

The sample size was estimated based on the value and desired precision of the proportions required for the study’s primary outcome measure (i.e., the proportion of patients “poorly”, “somewhat”, and “controlled” as assessed by the ACT). Using normative data for asthma control per the ACT in a US asthma patient population, it was estimated that approximately 60% of US patients with asthma are “controlled”^36^. Assuming a proportion of 50% of patients in the controlled and uncontrolled groups, a final target sample size of *n* = 385 assured a 95% confidence interval (CI) of having a precision of ±0.05 or better for all proportions observed. Based on an estimated 20% survey response rate (calculated as per American Association for Public Opinion Research (AAPOR) formulas^37^) and 15% attrition in the 18-month claims observation period, a sampling frame of 2250 patients (with 750 each in low-, medium-, and high-ICS-dose strata) was estimated to reach the minimum target final evaluable sample size.

Two analytic cohorts were created to address study objectives. The overall study cohort comprised participants with survey data and claims data for the 12-month baseline period. The follow-up study cohort included only the subset of patients who were also continuously enrolled for the 6-month period following the survey.

To identify risk factors associated with suboptimal control, two multivariate analyses were conducted based on control measured by the ACT and by the ACQ-6 in the overall study cohort. Factors associated with suboptimal asthma control as measured by the ACT were determined by a generalized linear model (binomial distribution, logit link), with suboptimal asthma control modeled as the binary dependent variable (poorly and somewhat controlled vs controlled). A wide range of covariates were considered for inclusion in the model, including age (continuous), sex (male or female), race (White, Black/African American or Other), marital status (married or living with a partner, or single, never married, separated, divorced and/or widowed), the highest level of education completed (2-year college degree or higher [yes/no]), place of residence (urban/city, suburban or rural), number of years with asthma (continuous), smoking status (non-smoker or ever-smoker), BMI category (calculated from a patient report of height and weight; patients were assigned to one four categories: normal [18.5–<25 kg/m^2^], overweight [25–<30 kg/m^2^], obese [≥30 kg/m^2^]), CCI score (0, 1, or ≥2), count of unique asthma-related comorbid conditions (continuous), PDC for ICS-containing medications (≥0.80 or <0.80), SABA utilization (>6 or ≤6 canister fills), and asthma-related outpatient visits (yes/no). Race was self-reported by respondents and was categorized as American Indian or Alaskan Native, Asian, Black, or African American, Native Hawaiian or Pacific Islander, White, and/or Other race. Ethnicity was self-reported by respondents and was categorized as Hispanic/Latino (yes vs no). For missing values, data were imputed using the most frequent (prevalent) values in order to retain the majority of patients in the regression analysis. Missing values for race were categorized as “White” and for urban/rural residence as “suburban”; missing values for other variables were not imputed. Factors associated with suboptimal asthma control as measured by the ACQ-6 were assessed by a proportional odds model (cumulative logit model), using the same dependent variable and list of covariates. In both ACT and ACQ-6 multivariate models, variables that demonstrated at least marginal statistical significance (*p* < 0.1) in univariate analyses were included as covariates in the final multivariate model, along with relevant clinical variables (age, sex, race, marital status, and level of education, CCI score, count of unique asthma-related comorbid conditions, BMI, and smoking status) regardless of their statistical significance.

To explore the impact of asthma control on future risk of asthma outcomes, a third multivariate model used asthma control as the independent variable to describe the future risk of “control” measured using exacerbations and high SABA use (a proxy for poor control) in the follow-up period, using data from the follow-up cohort. Factors associated with the composite measure of any asthma exacerbation or high SABA use (>4 SABA canister fills per year, i.e., >2 SABA canister fills during the 6-month follow-up period) in the follow-up period were determined using a generalized linear model (binomial distribution, logit link), developed in a stepwise manner adjusting for four sets of covariates of poorly/somewhat controlled asthma: ACT suboptimal control (yes/no), demographics (age [continuous], the highest level of education [2-year college degree or higher (yes/no)]), comorbidities (non-smoker or ever-smoker, BMI category [<30 or ≥30 kg/m^2^]), and ICS/LABA dose category (low, medium, or high).

Analyses were conducted using the SAS version 9.4 statistical software package (SAS Institute Inc., Cary, NC, USA). Study outcomes were analyzed descriptively unless otherwise specified.

### Reporting summary

Further information on research design is available in the Nature Research Reporting Summary linked to this article.

## Supplementary information

Supplementary Material File

Reporting Summary

## Data Availability

Information on GSK’s data-sharing commitments and requesting access to anonymized individual participant data and associated documents from GSK-sponsored studies can be found at www.clinicalstudydatarequest.com. The data reported in this publication are contained in a database owned by Optum, which contains proprietary elements. Therefore, it cannot be broadly disclosed or made publicly available at this time. The disclosure of this data to third parties assumes certain data security and privacy protocols are in place and that the third parties have executed Optum’s standard license agreement, which includes restrictive covenants governing the use of the data.
