# Supplementary material for: Suboptimally controlled asthma in patients treated with inhaled ICS/LABA: prevalence, risk factors, and outcomes
Source: NPJ Prim Care Respir Med. 2023 May 8;33:19. doi: 10.1038/s41533-023-00336-9 (PMC10167343; doi:10.1038/s41533-023-00336-9)

Supplementary materials

Supplementary Table 1. Patient demographics and clinical characteristics during the baseline period according to ACQ-6-assessed asthma control[1]

Overall survey cohort

	Overall[2] N=428	ACQ-6-assessed level of asthma control		
		Uncontrolled n=130	Partially controlled n=108	Controlled n=190
**Age (years)[3], mean (SD)**	49.8 (12.0)	49.4 (11.2)	49.2 (13.6)	50.4 (11.6)
**Female, n (%)**	286 (66.8)	90 (69.2)	65 (60.2)	131 (69.0)
**Race, n (%)**				
American Indian or Alaskan Native	7 (1.6)	4 (3.1)	2 (1.9)	1 (0.5)
Asian	4 (0.9)	0 (0)	2 (1.9)	2 (1.1)
Black or African American	26 (6.1)	12 (9.2)	4 (3.7)	10 (5.3)
Native Hawaiian or Pacific Islander	2 (0.5)	0 (0)	0 (0)	2 (1.1)
White	378 (88.3)	111 (85.4)	99 (91.7)	168 (88.4)
Other race	19 (4.4)	9 (6.9)	2 (1.9)	8 (4.2)
Missing	2 (0.5)	0 (0)	0 (0)	2 (1.1)
**Marital status, n (%)**				
Single, never married	58 (13.6)	16 (12.3)	20 (18.5)	22 (11.6)
Living with partner	30 (7.0)	7 (5.4)	9 (8.3)	14 (7.4)
Separated	6 (1.4)	2 (1.5)	0 (0)	4 (2.1)
Married	288 (67.3)	91 (70.0)	62 (57.4)	135 (71.1)
Divorced	39 (9.1)	10 (7.7)	15 (13.9)	14 (7.4)
Widowed	7 (1.6)	4 (3.1)	2 (1.9)	1 (0.5)

**Highest level of education completed, n (%)**				
Some college but no degree or lower	144 (33.7)	56 (43.1)	35 (32.7)	53 (27.9)
2-year college or higher	283 (66.3)	74 (56.9)	72 (67.3)	137 (72.1)
**Place of residence, n (%)**				
Urban/city	128 (30.1)	38 (29.7)	35 (32.4)	55 (29.0)
Suburban	224 (52.6)	57 (44.5)	59 (54.6)	108 (56.8)
Rural	74 (17.4)	33 (25.8)	14 (13.0)	27 (14.2)
**Age (years) at asthma diagnosis, mean (SD)**	*n=424*<br>23.9 (18.2)	*n=130*<br>24.8 (18.7)	*n=104*<br>23.8 (18.6)	*n=190*<br>23.3 (17.8)

[1]Defined as 12 months up to and including survey (index) date. Please refer to Table S2 for information on sources of specific data (patient survey and claims analysis); [2]overall study cohort: patients with survey data and claims data for the 12-month baseline period; [3]claims-based age calculated as of 2019.

ACQ-6, Asthma Control Questionnaire-6; SD, standard deviation.

Supplementary Table 2. Mini-AQLQ and EQ-5D-3L domains by ACT Level of asthma control

	Overall[1] N=428	ACT-assessed level of asthma control		
		Poorly controlled n=62	Somewhat controlled n=94	Controlled n=272
**AQLQ**				
Symptoms domain, mean (SD)	5.4 (1.2)	3.7 (0.9)	4.7 (0.8)	6.0 (0.8)
Activity limitation domain, mean (SD)	5.9 (1.2)	4.4 (1.3)	5.5 (1.1)	6.4 (0.8)
Emotional domain, mean (SD)	5.2 (1.7)	3.5 (1.5)	4.5 (1.6)	5.9 (1.3)
Environmental stimuli domain, mean (SD)	4.6 (1.6)	3.4 (1.4)	4.1 (1.5)	5.0 (1.5)
**EQ-5D-3L**				
**Mobility**				
I have no problems in walking about	379 (88.8)	46 (74.2)	80 (85.1)	253 (93.4)
I have some problems in walking about	48 (11.2)	16 (25.8)	14 (14.9)	18 (6.6)
I am confined to bed	0 (0)	0 (0)	0 (0)	0 (0)
**Self-care**				
I have no problems with self-care	422 (99.1)	58 (95.1)	93 (98.9)	271 (100)
I have some problems washing or dressing myself	4 (0.9)	3 (4.9)	1 (1.1)	0 (0)
I am unable to wash or dress myself	0 (0)	0 (0)	0 (0)	0 (0)
**Usual activities**				
I have no problems with performing my usual activities	352 (82.6)	35 (56.5)	71 (75.5)	246 (91.1)
I have some problems with performing my usual activities	74 (17.4)	27 (43.6)	23 (24.5)	24 (8.9)

I am unable to perform my usual activities	0 (0)	0 (0)	0 (0)	0 (0)
**Pain/discomfort**				
I have no pain or discomfort	265 (62.2)	22 (35.5)	46 (49.5)	197 (72.7)
I have moderate pain or discomfort	147 (34.5)	36 (58.1)	44 (47.3)	67 (24.7)
I have extreme pain or discomfort	14 (3.3)	4 (6.5)	3 (3.2)	7 (2.6)
**Anxiety/depression**				
I am not anxious or depressed	276 (64.6)	35 (56.5)	47 (50.0)	194 (71.6)
I am moderately anxious or depressed	138 (32.3)	23 (37.1)	45 (47.9)	70 (25.8)
I am extremely anxious or depressed	13 (3.0)	4 (6.5)	2 (2.1)	7 (2.6)

[1]Overall study cohort: patients with survey data and claims data for the 12-month baseline period.

ACT, Asthma Control Test; AQLQ, Asthma Quality of Life Questionnaire; EQ-5D-3L, EuroQol Group 5 Dimension Health Status Measure, 3-level; SD, standard deviation.

Supplementary Table 3. Categorization of low, medium, and high ICS/LABA cohorts based on GINA guidelines

**Drug**	**Daily dose (mcg)**		
	Low	Medium	High
Beclometasone dipropionate (CFC)[1]	200–500	>500–1000	>1000
Beclometasone dipropionate (HFA)	100–200	>200–400	>400
Budesonide (DPI)	200–400	>400–800	>800
Ciclesonide (HFA)	80–160	>160–320	>320
Fluticasone furoate (DPI)	100	n/a	200
Fluticasone propionate (DPI)	100–250	>250–500	>500
Fluticasone propionate (HFA)	100–250	>250–500	>500
Mometasone furoate	110–220	>220–440	>440
Triamcinolone acetonide	400–1000	>1000–2000	>2000

[1]Beclometasone dipropionate CFC is included for comparison with older literature.

CFC, chlorofluorocarbon propellant; DPI, dry powder inhaler; GINA, Global Initiative for Asthma; HFA, hydrofluoroalkane propellant; n/a, not applicable.

Supplementary Table 4. Study measure by data source and observation period

**Analytic measure**	**Patient survey**	**Baseline claims (12 months)**	**Follow-up claims (6 months)**
Asthma control – ACT and ACQ	X		
Asthma-related QoL – mini-AQLQ	X		
General health status – EQ-5D-3L	X		
Asthma treatment dispensings, maintenance and rescue (count)		X	X
Asthma treatment dispensings by class (count)		X	X
Adherence to asthma maintenance treatment(s) (PDC, MPR)		X	X
Medication burden – overall, asthma-related, high vs low SABA use		X	X
All-cause and asthma-related health care utilization		X	X
All-cause and asthma-related health care costs		X	X
Quan-Charlson comorbidity score		X	
Asthma-related comorbid conditions		X	X
Asthma exacerbations		X	X
Asthma Medication Ratio		X	X
Screening question: self-reported health care provider diagnosis of asthma	X		
Self-reported asthma history (age at asthma onset, age began using maintenance treatment, seasonal impact, health care provider diagnosis of allergies/allergic rhinitis)	X		
Clinical characteristics (height, weight, smoking status, pack years)	X		

Demographic and sociodemographic characteristics (age, sex, race, ethnicity, education, urban/suburban/rural location, household income)	X		

ACQ-6, Asthma Control Questionnaire-6; ACT, Asthma Control Test; EQ-5D-3L, EuroQol Group 5 Dimension Health Status Measure, 3-level; FDC, fixed-dose combination; mini-AQLQ, Asthma Quality of Life Questionnaire, short version; MPR, medication possession ratio; PDC, proportion of days covered; QoL, quality of life; SABA, short-acting β_2-agonist.

Supplementary Table 5. Categorization of ICS/LABA dose category based on the latest claim for an ICS/LABA medication prior to completing the survey

**Medication**	**Daily dose (mcg)**		
	Low	Medium	High
Advair Diskus	≤250	>250–500	>500
Advair HFA	≤114	>114–229	>229
AirDuo RespiClick	≤110	>110–463	>463
Breo	≤199	n/a	>199
Dulera	n/a	≤199	>199
Symbicort	≤159	>159	n/a

HFA, hydrofluoroalkane propellant; ICS, inhaled corticosteroid; LABA, long-acting $ß_2$-agonist; n/a, not applicable.

Supplementary Figure 1. Patient flow and attrition

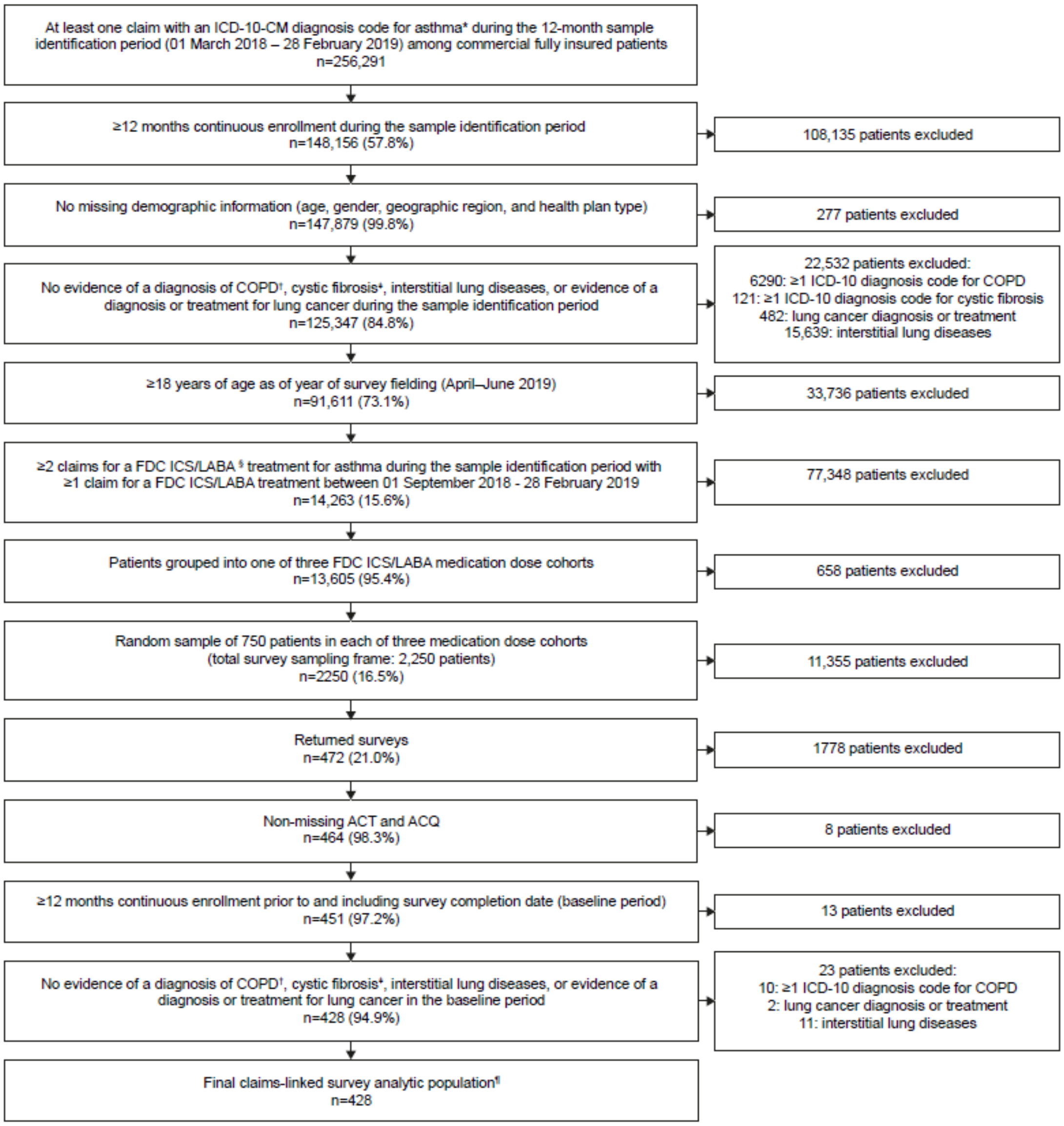

*Asthma ICD-10 diagnosis codes: J4520, J4521, J4522, J4530, J4531, J4532, J4540, J4541, J4542, J4550, J4551, J4552, J45901, J45902, J45909, J45990, J45991, J45998; †COPD ICD-10 diagnosis codes: J41, J410, J411, J418, J42, J43, J430, J431, J432, J438, J439, J44, J440, J441, J449; ‡cystic fibrosis ICD-10 diagnosis codes: E840, E8411, E8419, E848, E849; §Advair DISKUS, Advair HFA, AirDuo RespiClick, Breo ELLIPTA, Dulera, and/or Symbicort; ¶following a 6 month claims lag, patients were removed from the analysis if they had evidence of COPD, cystic fibrosis, interstitial lung diseases, lung cancer

(diagnosis or treatment), or if they had evidence of triple therapy during the 12 month baseline period. Continuous enrollment in the health plan was also re-assessed, and patients having <12 months of continuous enrollment in the baseline were removed from the analysis.

ACQ-6, Asthma Control Questionnaire-6; ACT, Asthma Control Test; COPD, chronic obstructive pulmonary disease; FDC, fixed-dose combination; ICD-10-CM, International Classification of Disease 10th edition Clinical Modification; ICS, inhaled corticosteroid; IQ, interquartile range; LABA, long-acting β_2-agonist.

Supplementary Figure 2. Concordance between asthma control measured by the ACT and the ACQ-6

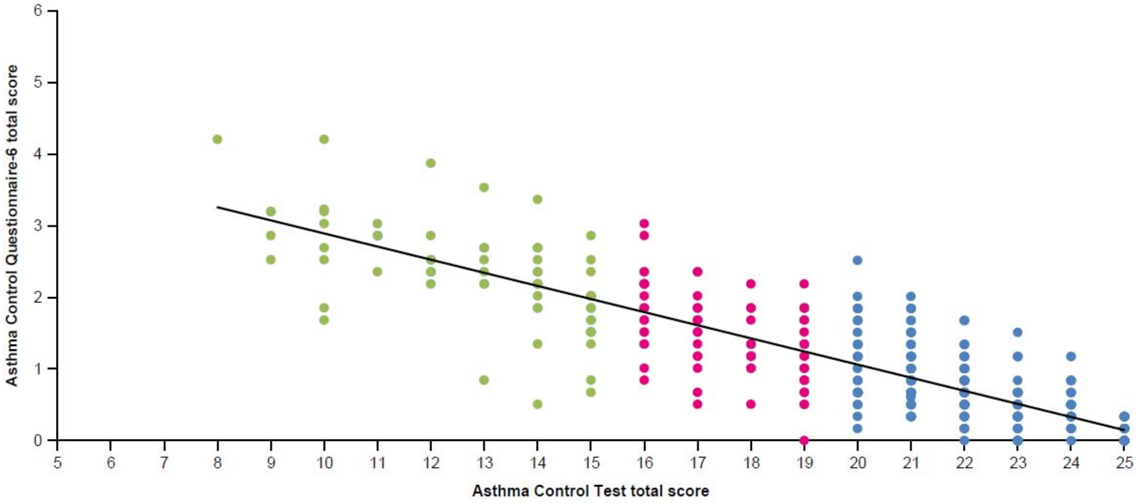

ACT total score ranges from 5 (poor asthma control) to 25 (complete control of asthma). The ACT results in three control levels: poorly controlled (score <16 [red]), somewhat controlled (score >16 –<19 [yellow]), and controlled (score ≥20–25 [green]). ACQ-6 total score ranges from 0 (totally controlled) to 6 (severely uncontrolled). The ACQ results in three control levels: controlled asthma (≤0.75), partially controlled asthma (>0.75 to <1.5), and uncontrolled asthma (≥1.50).

ACQ-6, Asthma Control Questionnaire-6; ACT, Asthma Control Test.

Supplementary Figure 3. Study design

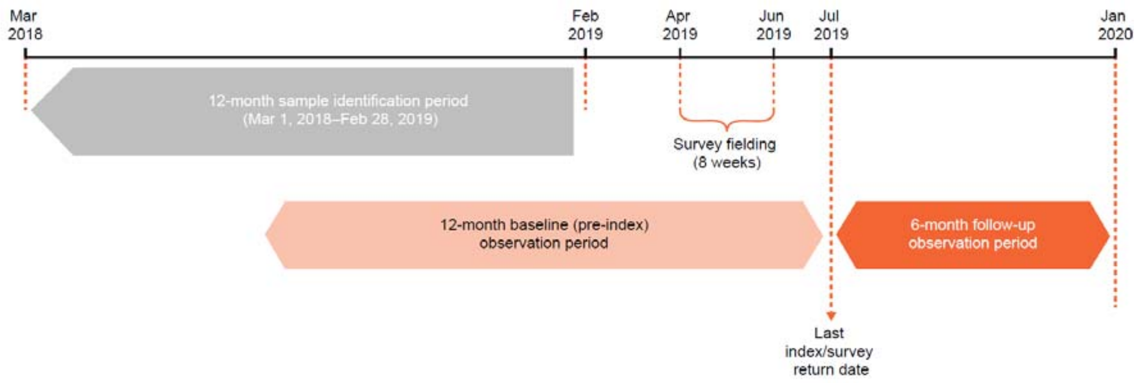

Supplement: Supplementary file 1 — Supplementary Material File [file 41533_2023_336_MOESM1_ESM.pdf]
