# Supplementary material for: Suboptimally controlled asthma in patients treated with inhaled ICS/LABA: prevalence, risk factors, and outcomes
Source: NPJ Prim Care Respir Med. 2023 May 8;33:19. doi: 10.1038/s41533-023-00336-9 (PMC10167343; doi:10.1038/s41533-023-00336-9)
Supplement: Supplementary file 2 — Reporting Summary [file 41533_2023_336_MOESM2_ESM.pdf]

nature portfolio

Corresponding author(s): Shiyuan Zhang

Last updated by author(s): Nov 7, 2022

Reporting Summary

Nature Portfolio wishes to improve the reproducibility of the work that we publish. This form provides structure for consistency and transparency in reporting. For further information on Nature Portfolio policies, see our Editorial Policies and the Editorial Policy Checklist.

Statistics

For all statistical analyses, confirm that the following items are present in the figure legend, table legend, main text, or Methods section.

n/a	Confirmed	
☐	☒	The exact sample size (*n*) for each experimental group/condition, given as a discrete number and unit of measurement
☒	☐	A statement on whether measurements were taken from distinct samples or whether the same sample was measured repeatedly
☐	☒	The statistical test(s) used AND whether they are one- or two-sided<br>*Only common tests should be described solely by name; describe more complex techniques in the Methods section.*
☐	☒	A description of all covariates tested
☐	☒	A description of any assumptions or corrections, such as tests of normality and adjustment for multiple comparisons
☐	☒	A full description of the statistical parameters including central tendency (e.g. means) or other basic estimates (e.g. regression coefficient) AND variation (e.g. standard deviation) or associated estimates of uncertainty (e.g. confidence intervals)
☒	☐	For null hypothesis testing, the test statistic (e.g. F, t, r) with confidence intervals, effect sizes, degrees of freedom and P value noted<br>*Give P values as exact values whenever suitable.*
☒	☐	For Bayesian analysis, information on the choice of priors and Markov chain Monte Carlo settings
☒	☐	For hierarchical and complex designs, identification of the appropriate level for tests and full reporting of outcomes
☒	☐	Estimates of effect sizes (e.g. Cohen's *d*, Pearson's *r*), indicating how they were calculated

Our web collection on statistics for biologists contains articles on many of the points above.

Software and code

Policy information about availability of computer code

Data collection	Optum Research Database
Data analysis	Analyses were conducted using the SAS version 9.4 statistical software package (SAS Institute Inc., Cary, NC, USA)

For manuscripts utilizing custom algorithms or software that are central to the research but not yet described in published literature, software must be made available to editors and reviewers. We strongly encourage code deposition in a community repository (e.g. GitHub). See the Nature Portfolio guidelines for submitting code & software for further information.

Data

Policy information about availability of data

All manuscripts must include a data availability statement. This statement should provide the following information, where applicable:

- Accession codes, unique identifiers, or web links for publicly available datasets
- A description of any restrictions on data availability
- For clinical datasets or third party data, please ensure that the statement adheres to our policy

Information on GSK's data sharing commitments and requesting access to anonymized individual participant data and associated documents from GSK-sponsored studies can be found at www.clinicalstudydatarequest.com. The data reported in this publication are contained in a database owned by Optum which contains proprietary elements. Therefore, it cannot be broadly disclosed or made publicly available at this time. The disclosure of this data to third parties assumes certain

data security and privacy protocols are in place and that the third parties have executed Optum's standard license agreement which includes restrictive covenants governing the use of the data.

Human research participants

Policy information about studies involving human research participants and Sex and Gender in Research.

Reporting on sex and gender	'A wide range of covariates were considered for inclusion in the model, including: age (continuous), sex (male or female)...'<br>'Claims data were used to assess geographic region, age and sex (if missing from the patient survey)...'<br>'The mean (standard deviation [SD]) patient age was 49.8 (12.0) years, 286 (66.8%) of patients were female...'
Population characteristics	'A wide range of covariates were considered for inclusion in the model, including: age (continuous), sex (male or female), race (White, Black/African American or Other), marital status (married or living with a partner, or single, never married, separated, divorced and/or widowed), highest level of education completed (2-year college degree or higher [yes/no]), place of residence (urban/city, suburban or rural), number of years with asthma (continuous), smoking status (non-smoker or ever-smoker), BMI category (calculated from patient report of height and weight; patients were assigned to one four categories: normal [18.5–<25 kg/m2], overweight [25–<30 kg/m2], obese [≥30 kg/m2]), CCI score (0, 1 or ≥2), count of unique asthma-related comorbid conditions (continuous), PDC for ICS-containing medications (≥0.80 or <0.80), SABA utilization (>6 or ≤6 canister fills), and asthma-related outpatient visits (yes/no).'
Recruitment	n/a.
Ethics oversight	The study was approved by the New England Institutional Review Board (IRB# 120190029) on 8 March 2019<br>'...after which eligible patients were invited to participate via mail following the Dillman method[32], with a survey fielding period of 8 weeks. A statement of informed consent was included; consent was implied by return of a completed survey.'

Note that full information on the approval of the study protocol must also be provided in the manuscript.

Field-specific reporting

Please select the one below that is the best fit for your research. If you are not sure, read the appropriate sections before making your selection.

☒ Life sciences ☐ Behavioural & social sciences ☐ Ecological, evolutionary & environmental sciences

For a reference copy of the document with all sections, see nature.com/documents/nr-reporting-summary-flat.pdf

Life sciences study design

All studies must disclose on these points even when the disclosure is negative.

Sample size	'The sample size was estimated based on the value and desired precision of the proportions required for the study's primary outcome measure (i.e., the proportion of patients 'poorly', 'somewhat', and 'controlled' as assessed by the ACT). Using normative data for asthma control per the ACT in a US asthma patient population, it was estimated that approximately 60% of US patients with asthma are 'controlled'[36]. Assuming a proportion of 50% of patients in the controlled and uncontrolled groups, a final target sample size of n=385 assured a 95% confidence interval (CI) of having a precision of ±0.05 or better for all proportions observed. Based on an estimated 20% survey response rate (calculated as per American Association for Public Opinion Research (AAPOR) formulas37) and 15% attrition in the 18-month claims observation period, a sampling frame of 2250 patients (with 750 each in low-, medium-, and high-ICS-dose strata) was estimated to reach the minimum target final evaluable sample size.'
Data exclusions	'Patients were excluded if they had an ICD-10-CM diagnosis code (i.e., ≥1 medical claim) for chronic obstructive pulmonary disease, cystic fibrosis, or interstitial lung diseases, or claims-based evidence of a diagnosis or treatment for lung cancer prior to or at the time of the survey fielding.'<br>'...Patients were excluded from the analytic sample if they did not return a complete survey (including evaluable ACT and ACQ-6), did not report a healthcare provider diagnosis of asthma, and/or did not report current asthma maintenance treatment. In addition to the claims criteria noted above, patients were also excluded from the final analytic sample if they did not have 12 months continuous enrollment prior to and including the survey completion date (baseline). Patients who disenrolled during the follow-up period were excluded from analyses that included the follow-up period.'
Replication	n/a.<br>'This was an observational study (HO-17-17252) of adults in the US with asthma treated with FDC ICS/LABA identified from the Optum Research Database (ORD)'
Randomization	'...Patients identified by these claims-based sample criteria were grouped into one of three FDC ICS/LABA medication dose cohorts (patients with claims for only low and/or low-medium daily dose treatments [low-dose cohort], medium and/or medium-high daily dose treatments [medium-dose cohort], or only high daily dose treatments [high-dose cohort]; Table S1). A random sample of 750 patients from each of these three medication dose cohorts was selected to receive the survey to ensure a range of asthma severity levels were included.'
Blinding	n/a.

Blinding	'This was an observational study (HO-17-17252) of adults in the US with asthma treated with FDC ICS/LABA identified from the Optum Research Database (ORD)'

Reporting for specific materials, systems and methods

We require information from authors about some types of materials, experimental systems and methods used in many studies. Here, indicate whether each material, system or method listed is relevant to your study. If you are not sure if a list item applies to your research, read the appropriate section before selecting a response.

Materials & experimental systems

n/a	Involved in the study
☒	☐ Antibodies
☒	☐ Eukaryotic cell lines
☒	☐ Palaeontology and archaeology
☒	☐ Animals and other organisms
☒	☐ Clinical data
☒	☐ Dual use research of concern

Methods

n/a	Involved in the study
☒	☐ ChIP-seq
☒	☐ Flow cytometry
☒	☐ MRI-based neuroimaging
